# Multistage carcinogenesis and the incidence of thyroid cancer in the US by sex, race, stage and histology

**DOI:** 10.1186/s12889-015-2108-4

**Published:** 2015-08-18

**Authors:** Rafael Meza, Joanne T. Chang

**Affiliations:** Department of Epidemiology, School of Public Health, University of Michigan, Ann Arbor, Michigan, SPH II, Room 5533, 1415 Washington Heights, Ann Arbor, MI 48109-2029 USA

## Abstract

**Background:**

Thyroid cancer has the fastest growing incidence in the US. However, the underlying causes are still under debate.

**Methods:**

We analyzed thyroid cancer incidence in the SEER-9 registry from 1973-2010 using multistage carcinogenesis and age-period-cohort models. Multistage models were used to investigate differences in initiation, promotion and malignant conversion rates of thyroid tumors by sex, race, stage, and histology. Models were adjusted for period and cohort trends to investigate the contributions of each factor, and determine whether birth- or diagnosis-year better correlate with observed incidence patterns.

**Results:**

Significant increases in thyroid cancer incidence by period or calendar-year were found for all sex, race, stage and histology combinations, particularly for localized cases (a 3- and 4-fold increase from 1973-2010 for females and males, respectively). Multistage analyses suggest that the 3-fold higher incidence in women could be explained by 1.5-fold higher initiation and promotion rates. Analyses by race suggest that the lower incidence in blacks can be attributed to lower promotion rates versus whites. Analysis by histology showed considerable decreases in follicular cancer incidence by birth-cohort since the early 1900s.

**Conclusions:**

Multistage modeling suggests that variations in thyroid cancer initiation and promotion can explain the observed differences in incidence by sex, race and histology. The consistent increases in incidence by calendar-year for all sex-race-histology-stage combinations suggest that the rise may be predominantly due to more intensive screening-diagnostics, although an environmental factor may be also at play. Our analyses constitute a first step towards the development of thyroid cancer natural history models.

**Electronic supplementary material:**

The online version of this article (doi:10.1186/s12889-015-2108-4) contains supplementary material, which is available to authorized users.

## Background

Thyroid cancer incidence worldwide has increased dramatically during the past three decades. Specifically, global age-standardized thyroid cancer incidence rates have increased 3-fold for both women and men since the 1970s, although with geographical variations [1]. Multiple descriptive studies have reported the upward trend of thyroid cancer [2–7]. However, the underlying causes are still under debate, with some attributing the increase to the widespread use of ionizing radiation therapy for head and neck benign conditions among children and adolescents back in the 1920-1950s [8, 9], while others attributing it to improvements in diagnostic tests and increase surveillance [10, 11].

The etiology of thyroid cancer is not fully understood, nonetheless it has been shown that gender and exposures to high-levels of ionizing radiation are major risk factors [12–14]. In terms of gender, thyroid cancer has become the fifth most common cancer among women in the United States [15], with a female to male ratio of 3:1. The gender imbalance has been attributed among other reasons to female hormonal and reproductive factors, which correlate with the age-specific rise in female thyroid cancer incidence around the age of menarche and the decrease or slow-down after menopause [1, 16]. With regards to ionizing radiation, previous studies have shown that atomic bomb survivors and children living in contaminated areas around Chernobyl in 1986 experienced particularly high rates of thyroid cancer. In addition, other studies have suggested that levels of iodine could be a risk factor for thyroid cancer. For example, patients with goiter, which suffer from iodine deficiency, have high rates of thyroid cancer [17]. There is also evidence that low levels of iodine in the diet associate with follicular thyroid cancers, whereas high levels are associated with papillary thyroid cancers [12].

Thyroid cancer has the fastest growing incidence in the US. Various authors have suggested that the rise in thyroid cancer incidence in the US is predominantly due to increases in surveillance and diagnostic improvements for detecting smaller tumors [2–4]. Other interpretations suggest that diagnostic scrutiny is not fully responsible for the increasing trends, but rather that there could be due to other factors like increases in obesity and changes in diet and physical activity [5–7] or exposures to environmental agents like radiation, Bisphenol A, and polybrominated biphenyl ethers [18–20]. Independently of the underlying reasons, thyroid cancer has become the fifth most common cancer in US women and has become significant public health issue.

Significant differences in thyroid cancer risk by race in the US have been reported [4, 21, 22], with whites having about twice the incidence than blacks. Healthcare access and socioeconomic status (SES) have been shown to be positively associated with thyroid cancer incidence, likely due to better detection and surveillance [4, 23]. Thus several authors have suggested that differences in SES by race may explain the lower thyroid cancer incidence in blacks [4, 23, 24]. However, it has been shown that differences in health care access between blacks and whites only explain partly the racial gap in thyroid cancer [24], and that increasing trends are similar between whites and blacks [16, 25]. Therefore other mechanisms, like racial variations in susceptibility and differences in exposures to environmental agents could be also responsible for the observed disparities [24, 26].

Several studies have previously examined thyroid cancer trends in SEER using descriptive analyses, joinpoint regression and APC models [2, 3, 16, 25]. Here we extend those analyses and complement them using multistage modeling that provides additional insights in terms of the biologicals mechanism of initiation, promotion and malignant conversion [27–33].

In this paper we investigated thyroid cancer incidence trends by sex, race, and stage in the US using multistage carcinogenesis models and age-period-cohort (APC) analysis. Multistage thyroid carcinogenesis models were used to investigate potential differences in initiation, promotion and malignant conversion rates by sex, race, stage and histology. Models were adjusted for period and birth-cohort trends to investigate the contributions of each of these factors, and to examine whether birth- or diagnosis-year better correlate with observed incidence patterns.

## Materials and methods

### Data sources

Thyroid cancer incidence data was obtained from the Surveillance, Epidemiology, and End Results (SEER)-9 registries for the years 1973–2010. We extracted reported thyroid cancer cases by sex, race, age, stage, histology and calendar year in the nine SEER geographic areas, which together represent an estimated 9.5 % of the U.S. population [34]. Thyroid cancer cases were coded using the International Classification of Diseases for Oncology, third edition (ICD-O-3) [35]. We restricted our analysis by histology to papillary (8050, 8052, 8130, 8260, 8340-8344, 8450, 8452) and follicular (8290, 8330-8332, 8335) types. Person-years by sex, race, age, and calendar year were obtained from the SEER registry. We analyzed combined thyroid cancer incidence to allow for race/gender comparisons, and also perform independent analyses by histology for all males and all females separately.

### Multistage model and age-period-cohort analyses

We performed likelihood-based analyses of the incidence of thyroid cancer in the SEER registries using APC models, where we replaced the non-specific age effects of traditional APC analyses with the hazard of multistage models. Secular trends, i.e., period and cohort effects, were modeled in the usual fashion as described below [28, 31, 32]. This approach constrains the age-effects parametrically and solves in principle the non-identifiability issues of APC models allowing us to estimate jointly the age, period and cohort trends [36]. Briefly, the thyroid cancer age-specific incidence at age a occurring in calendar year j is modeled as:

$$ {\mathit{\mathsf{h}}}_{\mathit{\mathsf{i}}\mathit{\mathsf{j}}}\left(\mathit{\mathsf{a}}\right)={\mathit{\mathsf{b}}}_{\mathit{\mathsf{i}}}{\mathit{\mathsf{c}}}_{\mathit{\mathsf{j}}}\mathit{\mathsf{h}}\left(\mathit{\mathsf{a}}\right); $$

where h(a) is the Two-Stage Clonal Expasion (TSCE) model hazard described below, c_j_ is a coefficient that adjusts for calendar year j, and the coefficient bi adjusts for birth cohort i (i = j-a , stratified in 5-year groups; <1890, 1890–1894,… ,1985-1990, and ≥1991). We used single ages from 0-84 and single calendar years from 1973-2010. We then fitted the model to the number of observed thyroid cancer cases (papillary and follicular combined) stratified by age and calendar-year. We obtained parameter estimates by maximizing the likelihood across all age-calendar strata assuming that the number of cases in each stratum is Poisson distributed with mean *N*_ij_*h_*ij*_*(a)*, where *N*_*ij*_ is the population at risk in age group *i* and birth cohort *j*, and *h*_*ij*_*(a)* is as defined above. Separate analyses for all sex, race, stage, and histology combinations were also performed. Multistage model analyses were done using the *Bhat* likelihood minimization package in R (R version 3.0.3).

In addition, we also fitted traditional APC models for comparison using the *Epi* package in R [37], and performed a joinpoint regression analysis using the statistical software Joinpoint, version 3.5 (Surveillance Research Program, US National Cancer Institute) [38] to characterize trends in age-adjusted incidence rates by sex, race, histology, and stage.

### Two-stage clonal expansion model

**Fig. 1:**
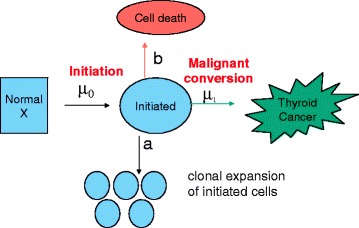
Two-stage clonal expansion (TSCE) carcinogenesis model

The TSCE model posits that cells initiated via a Poisson process undergo clonal expansion and malignant conversion via a birth–death–mutation process, and is based on the initiation-promotion-malignant conversion paradigm in carcinogenesis. The details of this model are presented elsewhere [27, 29]. Although the TSCE model is a simplification of the carcinogenesis and does not necessarily incorporate current knowledge about the natural history of thyroid cancer, it does capture the main aspects of tumor initiation-promotion and malignant conversion and thus has been used to analyze the temporal trends of a variety of cancers. In particular, this model and its generalizations have been used to analyze the incidence of a variety of cancers in SEER including colorectal cancer [28, 31, 33] esophageal cancer [30, 36], mesothelioma [32], and pancreatic cancer [31]. The TSCE model has four biological parameters: the rate of initiation, *μ*_*0*_*X*, the rate of division, *a*, and death, *b*, of initiated cells, and the rate of malignant conversion, *μ*_*1*_. Figure 1 shows a schematic representation of the TSCE model. Not all four parameters can be estimated from cancer incidence data alone. We estimated three identifiable parameters as described below. With constant parameters, the hazard function for this model takes the following form:

$$ h(t)=\frac{\mu_0X}{a}pq\frac{e^{- qt}-{e}^{- pt}}{q{e}^{- pt}-p{e}^{- qt}} $$

where *p* and *q* are the roots of a quadratic equation, with *p + q = -g = -(a - b-μ*_*1*_*)* and p*q = a*μ_*1*_. We estimated *p(*≈*-g)*, *q*, and *r = μ*_*0*_*X/a* ; which comprise a set of identifiable parameters. Note that *p* is roughly the net rate of proliferation of initiated cells (since *μ*_*1*_ is a mutation rate and thus much smaller than *a* and *b* ), *q ~ μ*_*1*_*/(1-b/a)*, and *r* is related to the rate of tumor initiation.

## Results

66,144 thyroid cancer cases were diagnosed from 1973 to 2010 in the SEER 9 registry areas, including 49,471 females and 16,673 males. The majority of cases occurred in whites (n = 40,379; 81.6 %) and papillary histological type (n = 53,809; 81.3 %). Regarding stage, 59.2 % of cases were diagnosed as localized, 32.7 % cases as regional, and 5.1 % cases as distant.

**Fig. 2:**
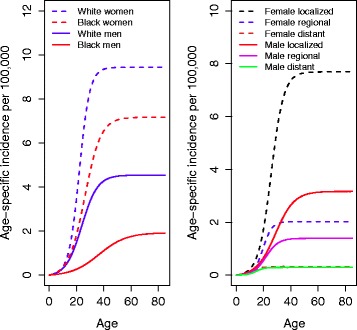
Thyroid cancer hazard (age-specific incidence) by race, gender and stage - TSCE-P-C thyroid cancer model

Figure 2 shows the estimated TSCE model hazards (age-specific incidence) by sex and race (all thyroid cancers combined) after removal of period and cohort effects (left- panel). In all cases the hazard starts at zero, grows slowly until becoming exponentially increasing and then slows down eventually reaching an asymptote (equal to -r*p). The age-specific thyroid cancer incidence is about 1.5-2.0 times higher in whites than blacks, and 2.5-3.0 times higher in women than in men. The right panel shows the estimated thyroid cancer TSCE model hazards by stage and sex (all races combined). We find that in both men and women the exponential increase in the age-specific incidence appears to start at younger ages for earlier stages, but that the asymptote is reached at younger ages for more advanced stages. So the range of exponential increase in age-specific risk is the longest for localized cancers and shortest for distant cancers.

Table 1 shows the estimated TSCE model biological parameters (*r*, *p* and *q*) by gender, race, stage and histology. As described above *r* is a measure of tumor initiation, -*p* represents the net cell proliferation of tumor cells, so it is a measure of premalignant tumor growth or promotion rate, and *q* is proportional to the malignant conversion rate. The table shows that the estimated initiation and promotion rates are consistently higher in women, whereas the malignant conversion rate is higher in men, and that promotion rates are about two-fold higher in whites versus blacks. Initiation rates decrease significantly by stage, explaining the higher age-specific incidence for earlier stages observed in Fig. 2. Whereas the estimated promotion rates, i.e., tumor growth rates, increase with stage, explaining the faster increase toward the asymptote for advanced stages in Fig. 2. In addition, there is no significant difference in biological parameters between papillary and follicular histologies.

**Table 1:** TSCE thyroid cancer incidence model parameter estimates

Parameter	*r*	95 % CI		*-p*	95 % CI		*q*	95 % CI	
Female	3.87E-04	3.46E-04	4.33E-04	2.49E-01	2.35E-01	2.49E-01	9.39E-04	7.73E-04	1.14E-03
Male	2.82E-04	2.24E-04	3.54E-04	1.68E-01	1.44E-01	1.95E-01	2.76E-03	2.02E-03	3.78E-03
White female	3.64E-04	3.22E-04	4.11E-04	2.59E-01	2.44E-01	2.76E-01	8.22E-04	6.62E-04	1.02E-03
White male	2.66E-04	2.08E-04	3.40E-04	1.71E-01	1.45E-01	2.00E-01	2.69E-03	1.91E-03	3.79E-03
Black female	4.26E-04	2.42E-04	7.49E-04	1.68E-01	1.22E-01	2.28E-01	1.97E-03	1.08E-03	3.57E-03
Black male	1.98E-04	7.90E-05	4.15E-04	9.73E-02	6.78E-02	1.37E-01	2.52E-03	1.01E-03	6.24E-03
Tumor stage									
Female									
Localize	3.58E-04	3.10E-04	4.13E-04	2.15E-01	2.00E-01	2.31E-01	8.51E-04	6.86E-04	1.06E-03
Regional	6.62E-05	5.36E-05	8.18E-05	3.05E-01	2.78E-01	3.34E-01	7.37E-04	5.09E-04	1.07E-03
Distant	9.52E-06	5.29E-06	1.71E-05	3.42E-01	2.13E-01	4.99E-01	3.45E-03	1.05E-03	1.13E-02
Tumor stage									
Male									
Localize	2.20E-04	1.58E-04	3.06E-04	1.44E-01	1.18E-01	1.74E-01	2.15E-03	1.51E-03	3.05E-03
Regional	6.43E-05	4.64E-05	8.91E-05	2.16E-01	1.75E-01	2.64E-01	1.86E-03	1.05E-03	3.31E-03
Distant	8.60E-06	3.94E-06	1.87E-05	3.37E-01	1.71E-01	5.57E-01	2.38E-03	3.40E-04	1.64E-02
Histology									
Female									
Papillary	2.84E-04	2.50E-04	3.23E-04	2.63E-01	2.47E-01	2.80E-01	7.70E-04	6.15E-04	9.62E-04
Follicular	5.44E-05	3.91E-05	7.57E-05	2.57E-01	2.14E-01	3.06E-01	1.09E-03	5.86E-04	2.03E-03
Histology									
Male									
Papillary	1.77E-04	1.40E-04	2.24E-04	2.00E-01	1.72E-01	2.30E-01	1.76E-03	1.19E-03	2.60E-03
Follicular	1.13E-04	8.68E-05	1.48E-04	1.50E-02	7.56E-01	1.50E-01	2.22E-03	1.38E-03	3.59E-03

To assess if year of diagnosis (period) or year of birth (cohort) is more relevant in determining thyroid cancer risk, we also fitted two-effect models (TSCE-Period or TSCE-Cohort) and compared their goodness-of-fit using the Akaike Information Criteria (AIC) [39]. Table 2 shows that in general the TSCE-Period models give a significantly better fit the data according to the AIC than the TSCE-Cohort models, suggesting that period or calendar-year better correlate with thyroid cancer incidence (with the possible exception of distant cancers, and cancers in black males). The table also shows the AICs for models that estimate both period and cohort effects simultaneously (TSCE-PC), which give the overall best fit and are therefore the preferred models.

**Table 2:** Akaike information criteria (AIC^a^) values for TSCE models relative to the TSCE/P model^b^

Relative AIC	TSCE/P	TSCE-P-C	TSCE/C
Females	0	-465.84	1215.04
Males	0	-177.24	145.14
White females	0	-540.56	974.3
White males	0	-157.3	113.076
Black females	0	-21.116	12.774
Black males	0	16.076	-18.528
Tumor stage (Females)			
Localized	0	-777.6	438.8
Regional	0	-88.16	463.04
Distant	0	-1.092	-4.488
Tumor stage (Males)			
Localized	0	-95.136	202.464
Regional	0	-55.4308	28.659
Distant	0	20.168	4.128

*TSCE* two-stage clonal expansion model, TSCE/P TSCE period; TSCE/C, TSCE cohort; TSCE-P-C, TSCE period-cohort

^a^ -2 × log(likelihood) + 2 × number of estimated parameters

^b^ Relative values that weight the goodness of fit of the model to empirical data. The lower the AIC, the better the model fit

**Fig. 3:**
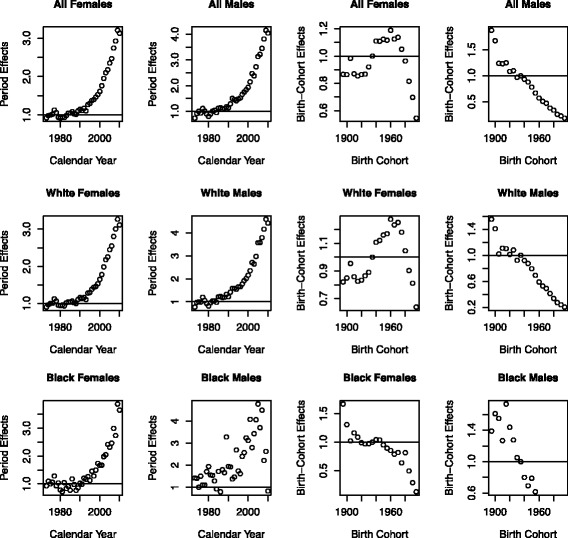
Thyroid cancer incidence period and cohort trends by gender and race – TSCE-P-C thyroid cancer model

Figure 3 shows the estimated period (calendar-year) and cohort (birth-year) effects from the final TSCE-PC models by race and sex (all thyroid cancers combined). Significant increases in thyroid cancer incidence by calendar-year (period) starting in the late 1980s are observed for all groups (~3-fold for females and ~ 4-fold for males). The increases by period are quite consistent by race for both females and males. The estimated birth-cohort effects are harder to interpret, but suggest a consistent decrease in incidence for more recent birth-cohorts, which is overshadowed by the significant increases by calendar-year.

**Fig. 4:**
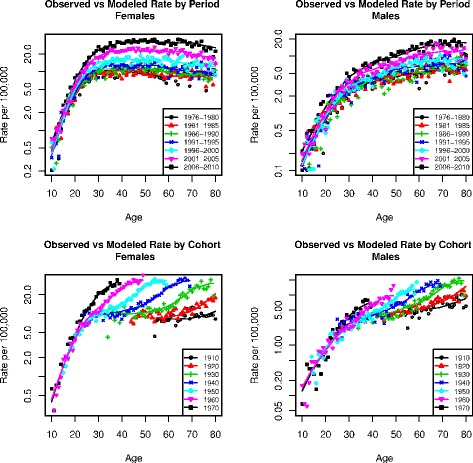
Observed versus modeled age-specific thyroid cancer rates by period and cohort

Figure 4 shows observed versus predicted age-specific thyroid incidence by gender for selected years and cohorts. The predicted curves are constructed by multiplying the estimated TSCE hazard, by the corresponding period and cohort effects. The figure shows that the models do capture the age and time trends observed in the data. Additional figures comparing the models by race and gender and observed data are shown in the supplementary material (Additional file 1: Figures S7 and S8).

**Fig. 5:**
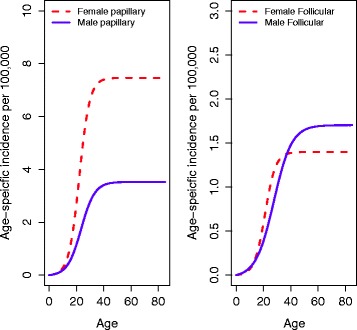
Thyroid cancer TSCE hazard by histology

**Fig. 6:**
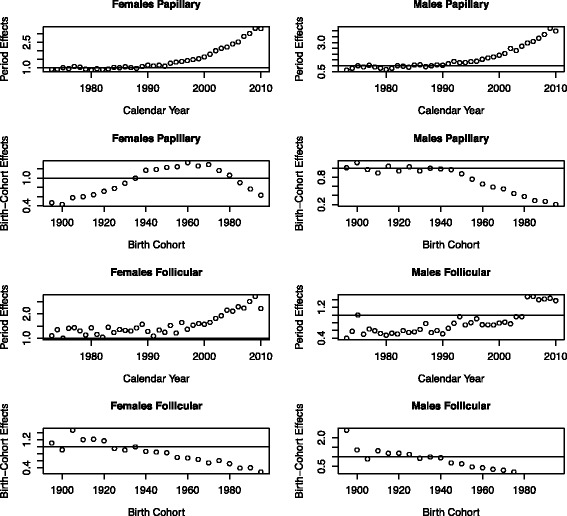
Thyroid cancer incidence period and cohort trends by histology and gender

Figure 5 shows the estimated hazards (age-specific incidence) by histology (papillary and follicular) after removal of period and cohort effects. The age-specific incidence for papillary histology is about 3.5-4 and 2 times higher than that of follicular histology in females and males, respectively, with the papillary incidence being considerably much higher in females versus males. We find that for both histologies the exponential increase in the age-specific incidence starts at a younger age in females. Figure 6 shows the estimated period and cohort trends by histological type. Significant increases in thyroid cancer incidence by calendar-year starting in the late 1980s are observed for both histologies, mimicking the trends found for all thyroid cancers. Cohort trends for papillary cancers by gender resemble those for all thyroid cancers, whereas for follicular cancer cohort trends are decreasing for cohorts starting since the early 1900s. Observed versus predicted thyroid cancer incidence figures by histology are shown in the supplemental material (Additional file 1: Figures S9 and S10).

Results of standard APC analyses for all thyroid cancers are also shown in the appendix. Overall, these show roughly consistent results with the multistage model analyses, particularly for the period trends, and suggest that period rather than cohort is more relevant in determining thyroid cancer risk. Joinpoint analysis results are also shown in the appendix and show consistent trends by calendar year as found with the multistage models.

## Discussion

In this paper we investigated thyroid cancer incidence trends by sex, race, stage and histology in the US using multistage carcinogenesis and age-period-cohort models. Our analyses suggested that period rather than cohort is more significant in determining thyroid cancer rates, with the possible exception of distant cancers, and that the increase of thyroid cancer by calendar-year is consistent for all sex, race, and histology combinations analyzed. These results together with the observation from joinpoint analyses (appendix) showing that the largest recent increases have occurred primarily for localized cancers suggest that the rising incidence may be predominantly due to more intensive surveillance and improved diagnostics. Nonetheless, the consistent calendar-year increases by race, which have occurred in presence of significant disparities in health care access by socioeconomic status and race [4, 23, 24], suggest that an environmental factor may be also at play.

Previous analyses have compared thyroid cancer incidence trends by sex, stage, and race using joinpoint and APC models [2, 3, 16, 25, 40]. Here we complement these with multistage modeling analyses, which allowed us to generate hypotheses about the biological mechanisms of thyroid cancer tumor initiation, promotion and malignant conversion. For instance, the multistage analyses show that the 3-fold higher thyroid cancer incidence in women versus men can be explained by 1.5-fold higher rates of initiation and promotion (premalignant tumor growth). These imply that women get 50 % more tumors and that those progress 50 % more rapidly to cancer than men’s. The lower thyroid cancer incidence in blacks can be attributed in part to lower tumor promotion rates versus whites. Sub-analyses of thyroid cancer incidence by stage reveal gradients of tumor initiation (localized > regional > distant) and promotion (localized < regional < distant) that suggest heterogeneity in aggressiveness from tumor onset with clear implications for the overdiagnosis of slow growing tumors under active screening and surveillance.

The estimated TSCE hazards (age-specific incidence) display a pattern where the asymptote is reached in middle age (roughly flat after age 40). This appears to be in contrast with previous analyses of thyroid cancer based on age-period-cohort models that have found age-specific patterns by period that decrease after age 40, or by cohort that increase until age 70 [40]. Our results are indeed consistent with these previous findings, once the TSCE hazards are multiplied by the corresponding estimated period and cohort effects as shown in Fig. 4 (and Additional file 1: Figures S7, S8, S9 and 10s). This suggests that a constant age-specific thyroid cancer risk from age 40-50 is consistent with the SEER data, and that the observed decreases in age-specific risk in different years (period) or increases in age-specific risk until late in life by cohort, might just be representing un-adjusted secular trends and not true age patterns in risk.

Analyses of thyroid cancer incidence by histological type (papillary vs follicular) show that interestingly estimated promotion rates do not seem to vary by histology, but that initiation of papillary cancers is about 6 and 1.5 times higher than that of follicular cancers in females and males, respectively. The higher initiation rates could be attributed to higher underlying mutation rates or to a higher number of susceptible cells. Interestingly, the estimated period effects do not vary much by histology, suggesting a common risk factor or an underlying cause behind the thyroid cancer increase with calendar year (potentially increased screening). However, the estimated birth-cohort effects do vary by histology, and show a decreasing trend for follicular cancers since the early 1900s possibly reflecting the reductions in risk due to iodization of salt in the US [41].

Our study has several limitations. First, in common with other analyses of cancer registry data, we were unable to assess exposures to relevant risk factors, such as dietary patterns and environmental exposures, in the underlying population and cancer cases. Nonetheless, the SEER registry allowed us to analyze trends in thyroid cancer incidence since the 1970s by sex, race, stage and histology. Second, our multistage models are clearly a simplification of the underlying biology of thyroid cancer incidence and neglect the contribution of relevant risk factors. As mentioned above, this is largely due to the lack of risk factor data from the SEER cancer registry. However, the age-period-cohort approach allowed us to control for secular trends in the estimation of the biological parameters. Moreover, the estimated TSCE models provide better fits to the data than more complex models that allow for additional carcinogenesis stages (data not shown), and the differences in biological parameters provide plausible explanations to the observed differences in age-specific thyroid cancer risk. Third, age-period-cohort models have an inherent non-identifiability problem that makes it impossible to estimate uniquely the period and cohort effects [42, 43]. However, replacing the age effects with the hazard of a multistage model resolves, at least in theory, the non-identifiability problem, allowing us to estimate uniquely the secular trends [36]. Finally, the smaller sample sizes for some demographic groups, like black males, or for specific histologies, like follicular cancer in men, preclude us from doing more detailed analyses.

## Conclusions

In summary, our analyses provide additional evidence that indicates that the rise in thyroid cancer incidence is likely predominantly due to more intensive screening-diagnostics, but also suggest that an environmental factor could be also at play. Given the recent evidence that indicates that current screening and imaging practices have led to significant levels of thyroid cancer overdiagnosis in the US [44, 45], there is a need to develop thyroid cancer natural history models to quantify the impact of such practices on observed thyroid cancer rates. Such models could be also used to investigate the potential impact of interventions to reduce thyroid cancer incidence and mortality and to predict the potential benefits (and harms) of changes in surveillance and screening practices on thyroid cancer outcomes [46, 47]. The models developed here constitute a first step in that direction [31, 33, 46].

## Additional file

Additional file 1: Table S1. Trends of thyroid cancer incidence by gender, race, histology and stage –Joinpoint analyses 1973-2010. **Figure S1.** Thyroid cancer incidence period and cohort trends by gender and stage – TSCE-P-C thyroid cancer model. **Figure S2**. Thyroid cancer age-adjusted incidence rates by gender, race and stage. **Table S2.** Akaike information criteria (AIC*) values for Age-Period-Cohort models relative to the AC model**. **Figure S3.** Age effects by race and sex (all cases)- Age-period-cohort models. **Figure S4.** Age-effects by tumor stage (all cases)- Age-period-cohort models. **Figure S5.** Thyroid cancer incidence period and cohort trends by gender and race – Age-period-cohort models. **Figure S6.** Thyroid cancer incidence period and cohort trends by gender and stage – Age-period-cohort models. **Figure S7.** Observed versus fitted thyroid cancer incidence by gender in Whites. **Figure S8.** Observed versus fitted thyroid cancer incidence by gender in Blacks. **Figure S9.**Observed versus fitted thyroid cancer incidence among papillary histology by gender. **Figure S10.** Observed versus fitted thyroid cancer incidence among follicular histology by gender. (PDF 1164 kb)

## Abbreviations

SES: Socioeconomic status
APC: Age-period-cohort
TSCE: Two-Stage Clonal Expansion
SEER: Surveillance, Epidemiology, and End Results
TSCE/P: TSCE-Period
TSCE/C: TSCE-Cohort
TSCE-P-C: TSCE-Period-Cohort
AIC: Akaike Information Criteria
ICD-O-3: International Classification of Diseases for Oncology, third edition
