# Supplementary material for: Multistage carcinogenesis and the incidence of thyroid cancer in the US by sex, race, stage and histology
Source: BMC Public Health. 2015 Aug 18;15:789. doi: 10.1186/s12889-015-2108-4 (PMC4539703; doi:10.1186/s12889-015-2108-4)

Multistage carcinogenesis and the incidence of thyroid cancer in the US by sex, race, stage and histology

Supplementary information

Rafael Meza and Joanne T. Chang

Table 1s. Trends of thyroid cancer incidence by gender, race, histology and stage –Joinpoint analyses 1973-2010

	Trend 1		Trend 2		Trend 3		Trend 4		Trend 5	
	Period 1	APC (95% CI)	Period 2	APC (95% CI)	Period 3	APC (95% CI)	Period 4	APC (95% CI)	Period 5	APC (95% CI)
Both Sexes										
All Races	1973-1977	5.5 (2.4,8.7)	1977-1980	-6.7 (-15.2, 2.6)	1980-1997	2.5 (2.1, 2.8)	1997-2010	6.5 (5.9, 7.0)		
White	1973-1977	6.1 (2.8, 9.4)	1977-1980	-6.1 (-14.8, 3.5)	1980-1996	2.6 (2.2, 3.0)	1996-2010	6.6 (6.1, 7.0)		
Black	1973-1989	-0.7 (-1.9, 0.6)	1989-2010	5.1 (4.2, 6.0)						
Other	1973-1996	-1.3 (-1.9, -0.6)	1996-2010	4.0 (2.6, 5.4)						
Men										
All Races	1973-1977	8.1 (2.2, 14.2)	1977-1980	-9.2 (-23.8, 8.2)	1980-1998	2.1 (1.5, 2.7)	1998-2010	5.6 (4.5, 6.7)		
White	1973-1977	8.7 (3.3, 14.4)	1977-1980	-9.3 (-22.7, 6.5)	1980-1998	2.6 (2.0, 3.2)	1998-2010	5.9 (4.9, 6.9)		
Black	1973-2010	2.5 (1.6, 3.5)								
Other	1973-1990	-4.8 (-6.4, -3.1)	1990-2010	1.6 (0.2, 3.0)						
Women										
All Races	1973-1977	5.1 (1.7, 8.6)	1977-1980	-5.5 (-14.8, 4.8)	1980-1996	2.5 (2.1, 3.0)	1996-2010	6.6 (6.0, 7.1)		
White	1973-1977	5.8 (2.1, 9.7)	1977-1980	-4.9 (-15.1, 6.6)	1980-1995	2.5 (2.0, 3.1)	1995-2010	6.8 (6.3, 7.3)		
Black	1973-1988	-1.3 (-2.7, 0.0)	1988-2010	5.3 (4.5, 6.1)						
Other	1973-1983	-3.7 (-5.7, -1.60	1983-1986	15.7 (-11.9, 51.9)	1986-1991	-7.2 (-14.9, 1.1)	1991-2010	3.3 (2.5, 4.2)		
Papillary	1973-1977	7.7 (3.9, 11.60	1977-1980	-6.6 (16.6, 4.5)	1980-1996	3.3 (2.8, 3.8)	1996-2010	6.9 (6.4, 7.5)		
Follicular	1973-1999	-0.4 (-0.8, 0.1)	1999-2010	3.3 (1.5, 5.1)						
Other	1973-2010	0.2 (-0.3, 0.6)								
Localized	1973-1975	15.8 (-2.1, 36.8)	1975-1990	-1.2 (-2.0, -0.4)	1990-2010	6.6 (6.1, 7.1)				
Regional	1973-1977	10.4 (6.1, 14.9)	1977-1981	-6.1 (-11.8, 0.0)	1981-1985	12.2 (5.4, 19.5)	1985-1999	1.7 (1.0, 2.4)	1999-2010	6.4 (5.5, 7.3)
Distant	1973-1986	-2.5 (-4.8, -0.2)	1986-2010	2.6 (1.6, 3.5)						

apc: annual percentage change

[a.] significant at $p<0.05$

Figure 1s. ***Thyroid cancer incidence period and cohort trends by gender and stage – TSCE-P-C thyroid cancer model***

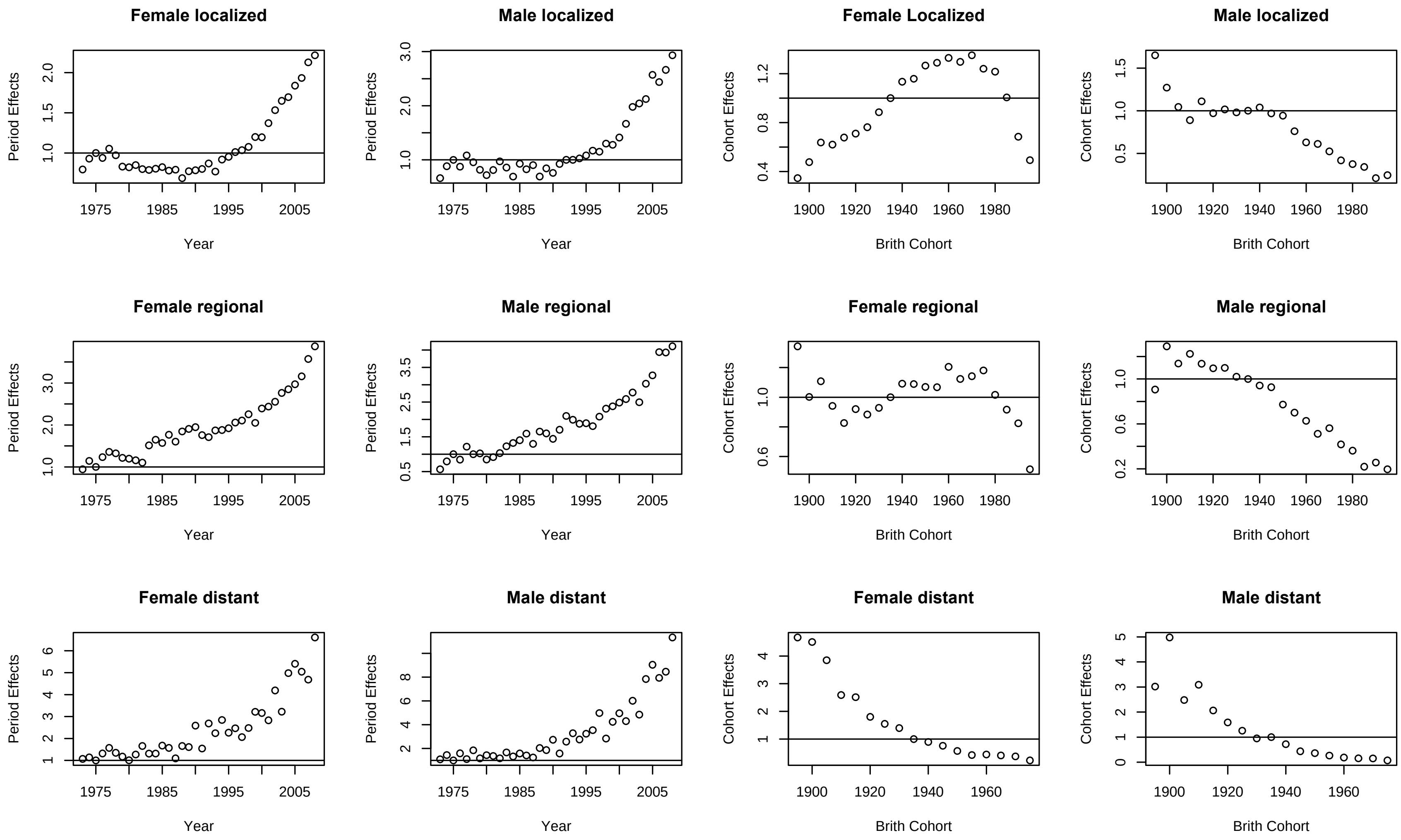

Figure 2s. Thyroid cancer age-adjusted incidence rates by gender, race and stage

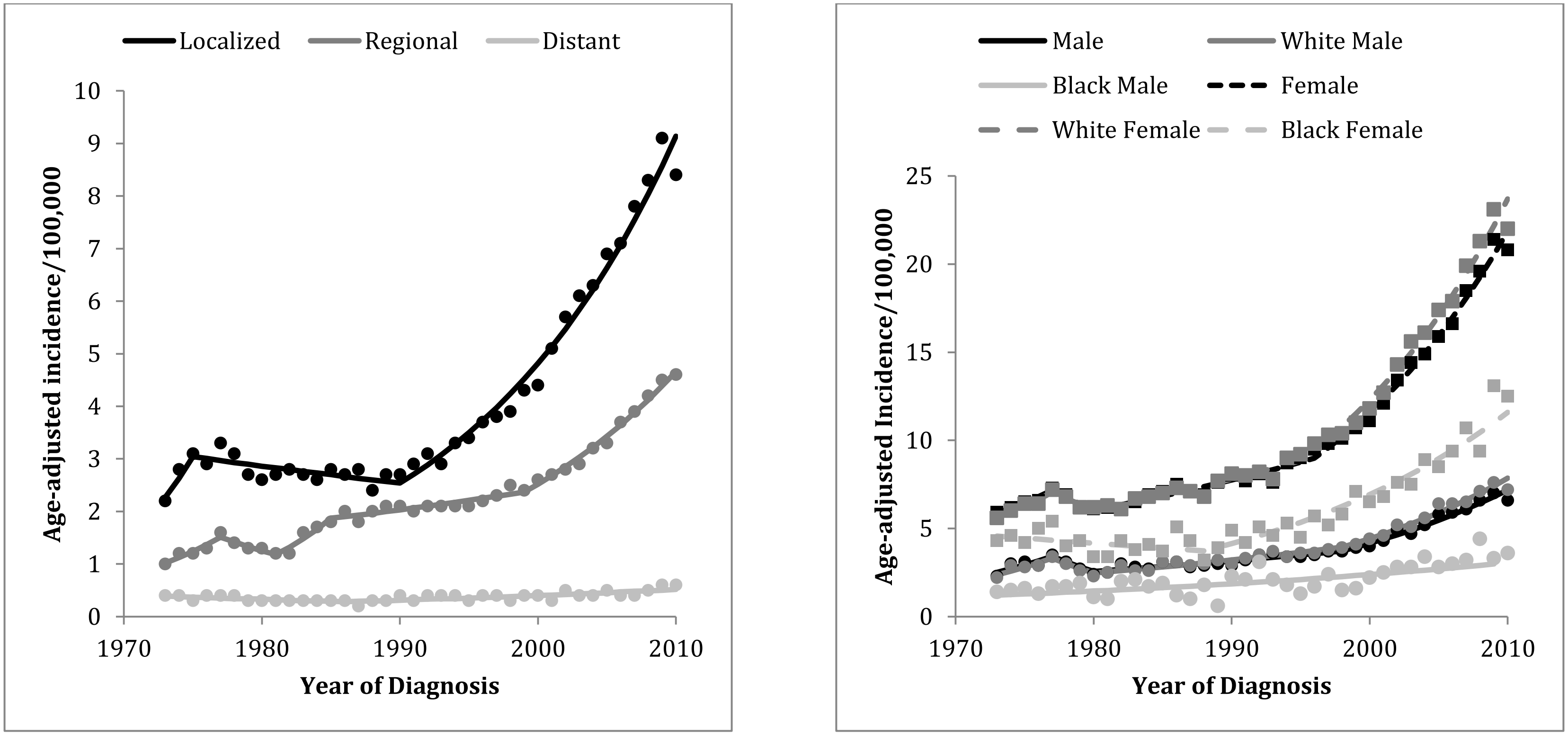

Table 2s. Akaike information criteria (AIC*) values for Age-Period-Cohort models relative to the AC model**

	AIC			Relative AIC **		
	AC	AP	AP-C	AC	AP	AP-C
Females	-174120.5	-174571.84	-174729.68	0	-451.34	-609.18
Males	-26887.74	-27012.24	-27054.12	0	-124.5	-166.38
White females	-128440.88	-128818.6	-128970.3	0	-377.72	-529.42
White males	-19117.082	-19195.702	-19237.898	0	-78.62	-120.816
Black females	3743.768	3732.542	3743.278	0	-11.226	-0.49
Black males	2964.93	2975.158	2995.378	0	10.228	30.448

AC, age-cohort; AP, age-period; AP-C, age-period-cohort.

* -2×log(likelihood)+2×number of estimated parameters.

** Relative values that weight the goodness of fit of the model to empirical data. The lower the AIC, the better the model fit.

Figure 3s. ***Age effects by race and sex (all cases)- Age-period-cohort models***

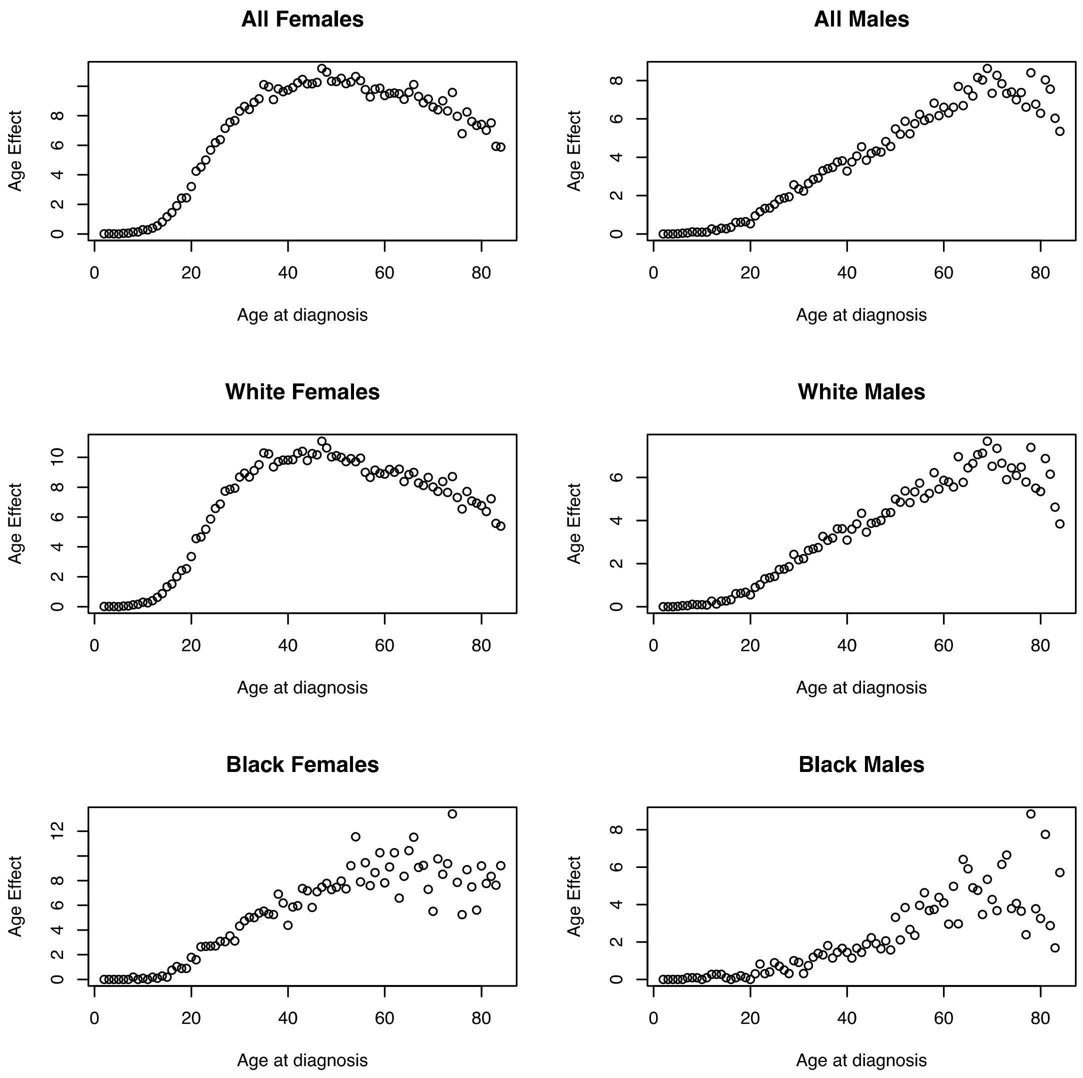

Figure 4s. ***Age-effects by tumor stage (all cases)- Age-period-cohort models***

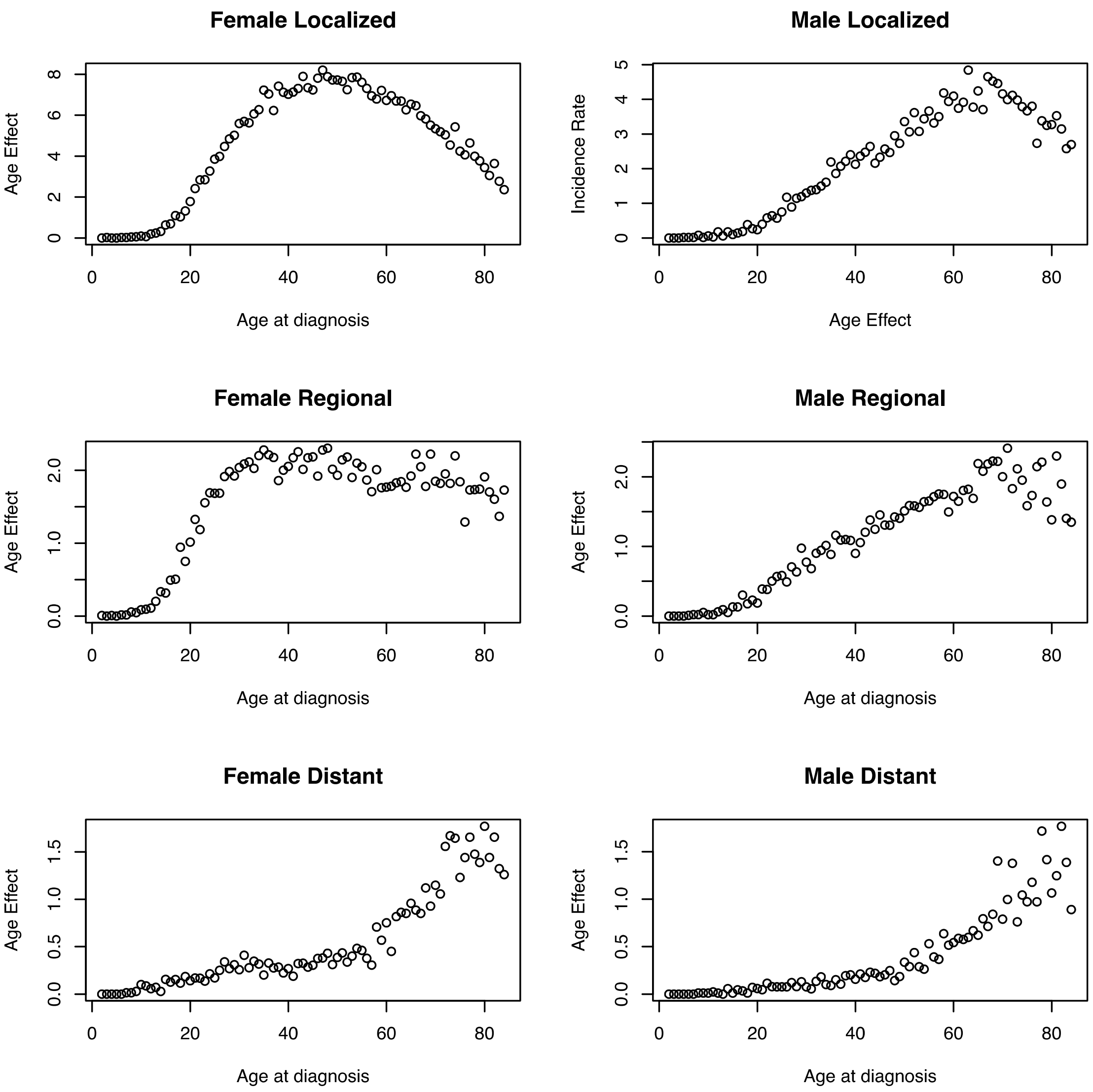

Figure 5s. ***Thyroid cancer incidence period and cohort trends by gender and race – Age-period-cohort models***

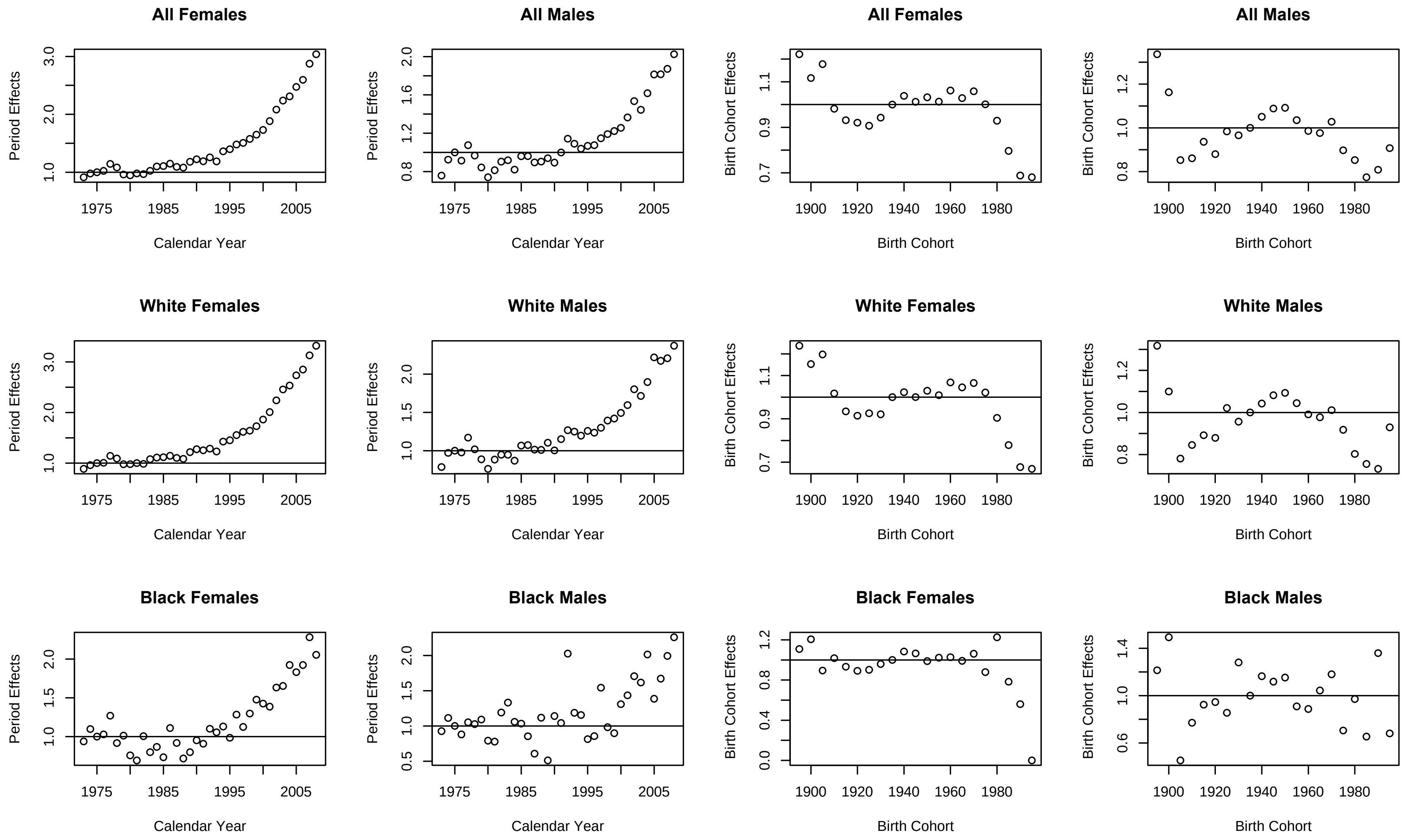

Figure 6s. ***Thyroid cancer incidence period and cohort trends by gender and stage – Age-period-cohort models***

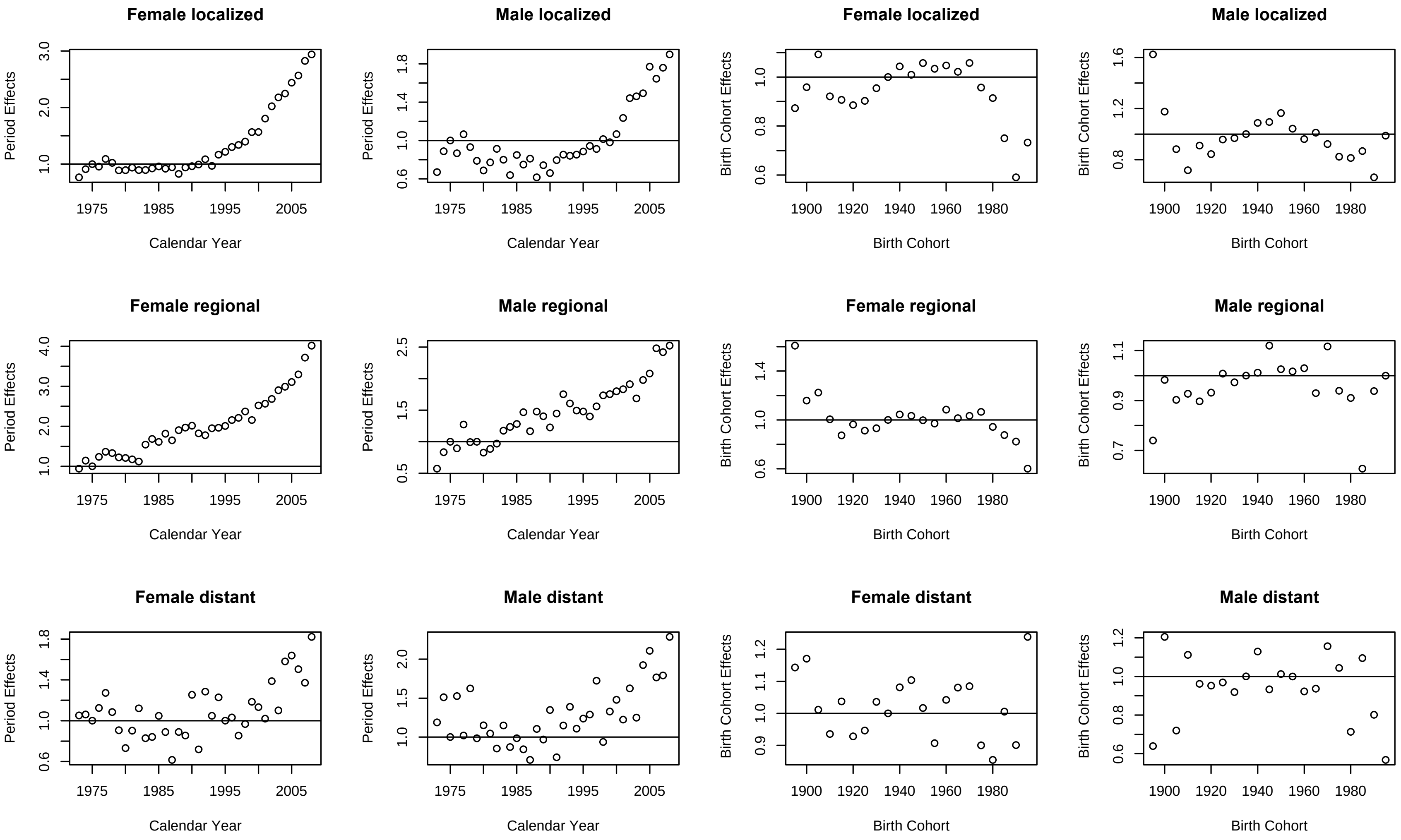

Figure 7s. ***Observed versus fitted thyroid cancer incidence by gender in Whites***

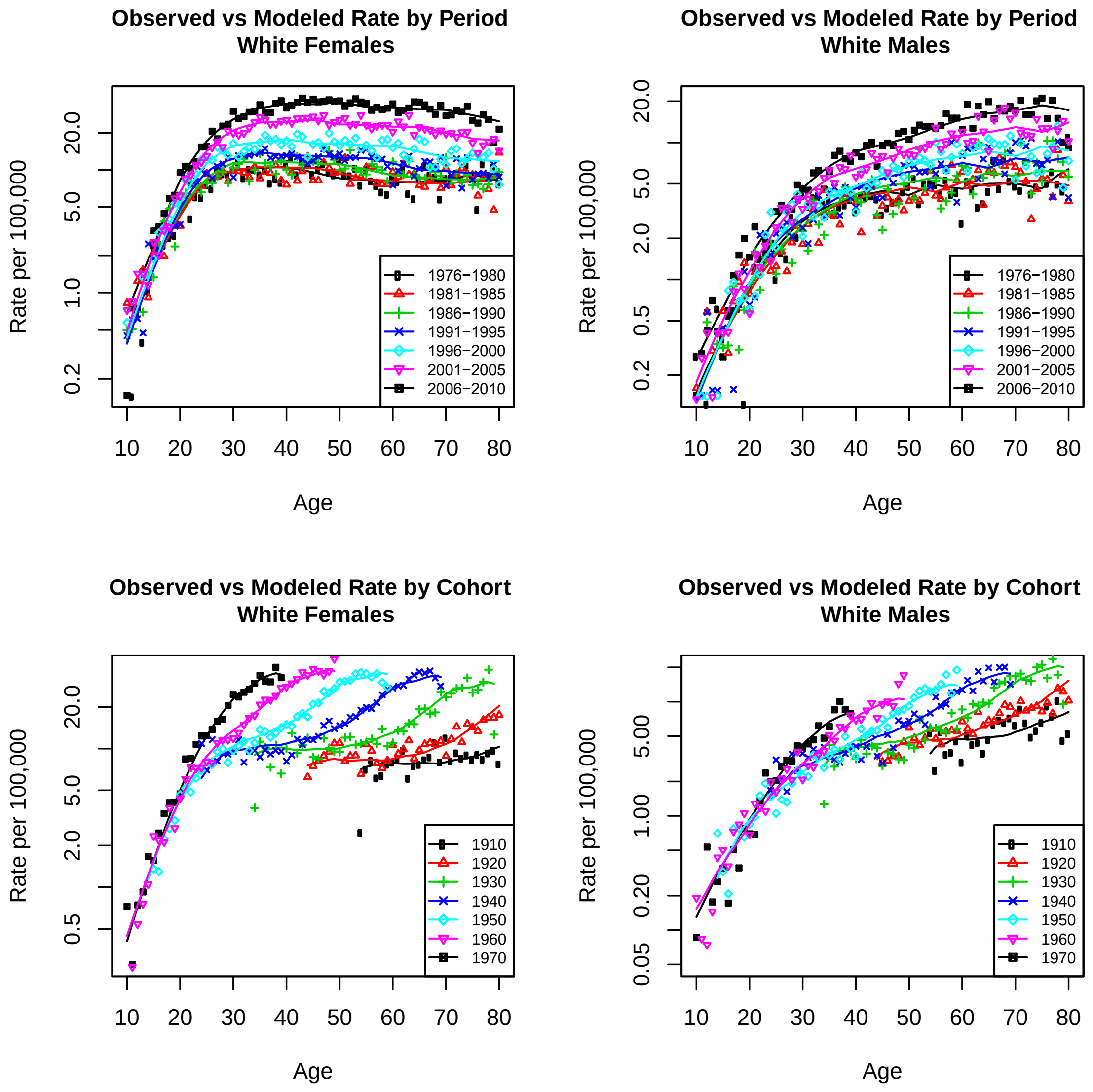

Figure 8s. ***Observed versus fitted thyroid cancer incidence by gender in Blacks***

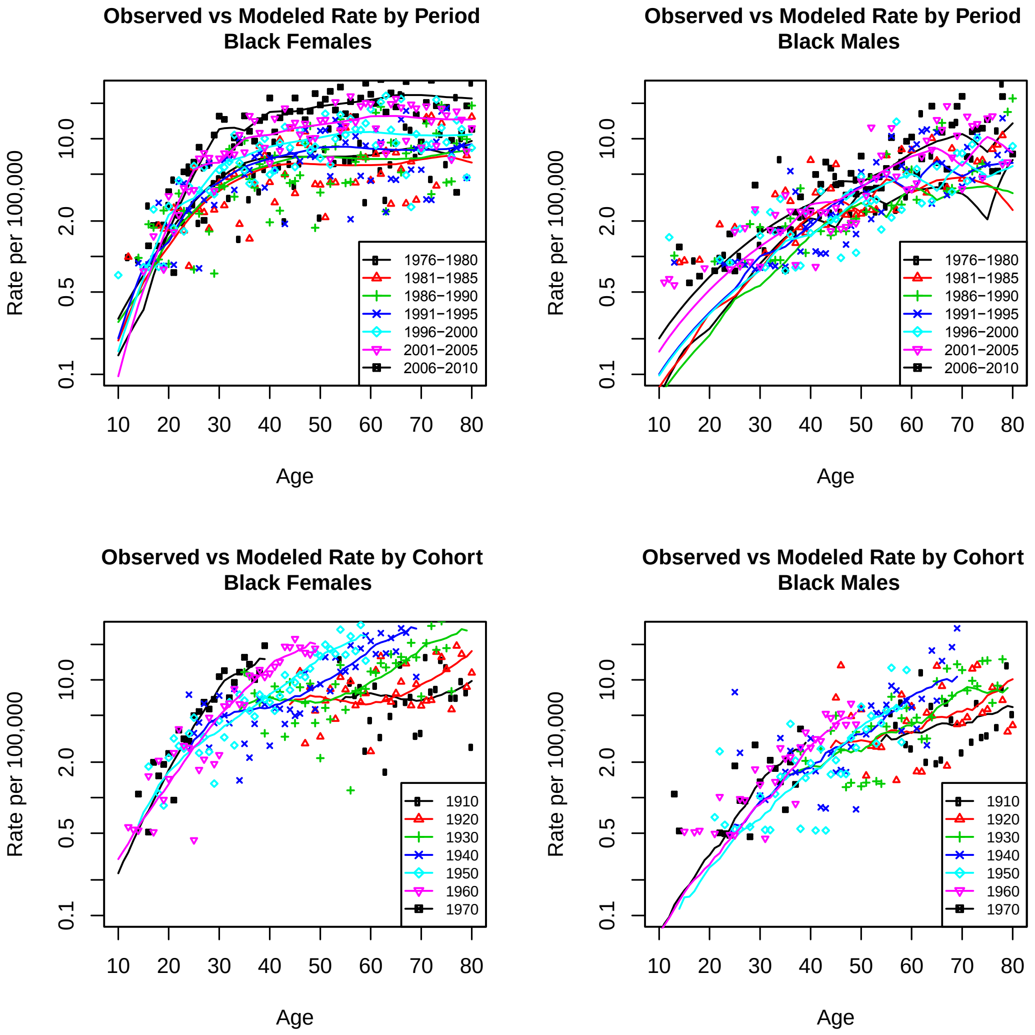

Figure 9s. ***Observed versus fitted thyroid cancer incidence among papillary histology by gender***

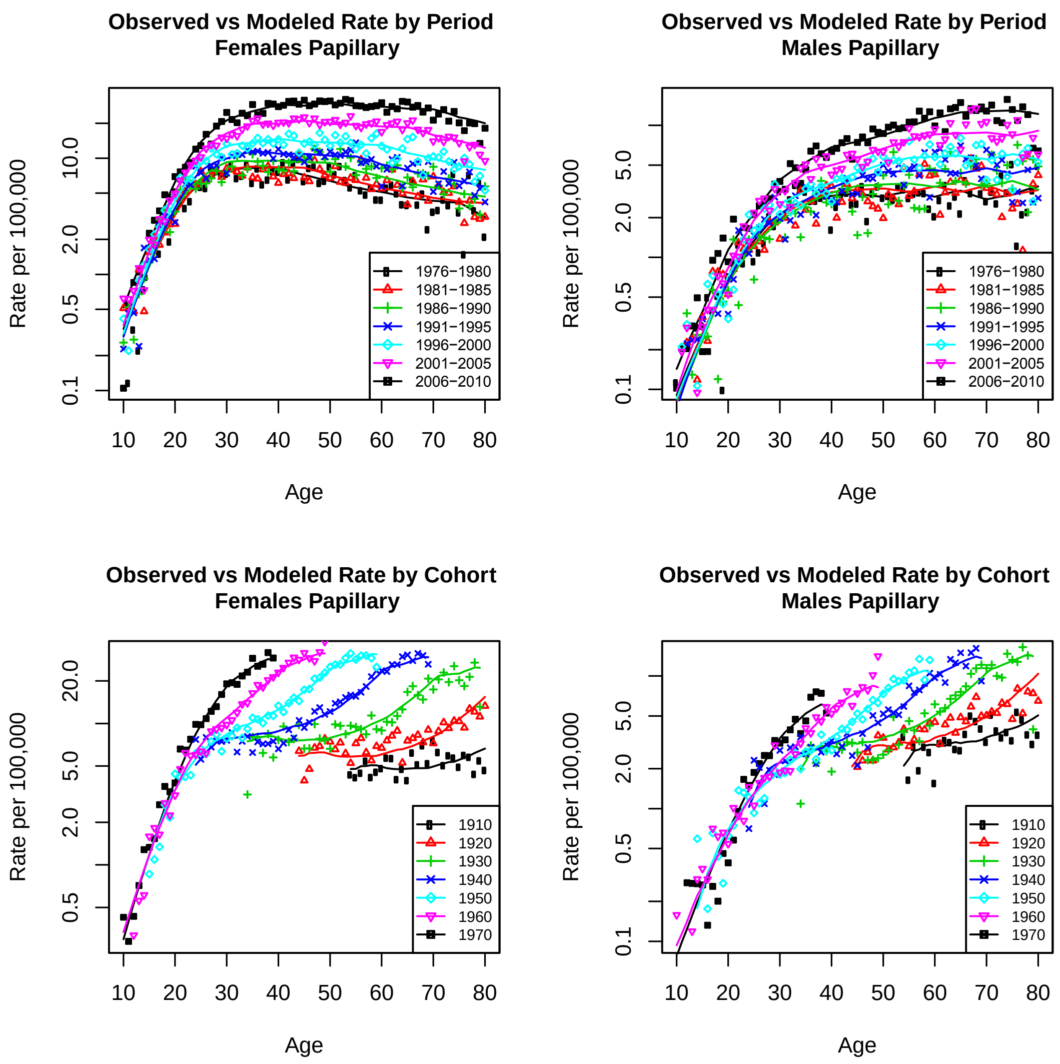

Figure 10s. ***Observed versus fitted thyroid cancer incidence among follicular histology by gender***

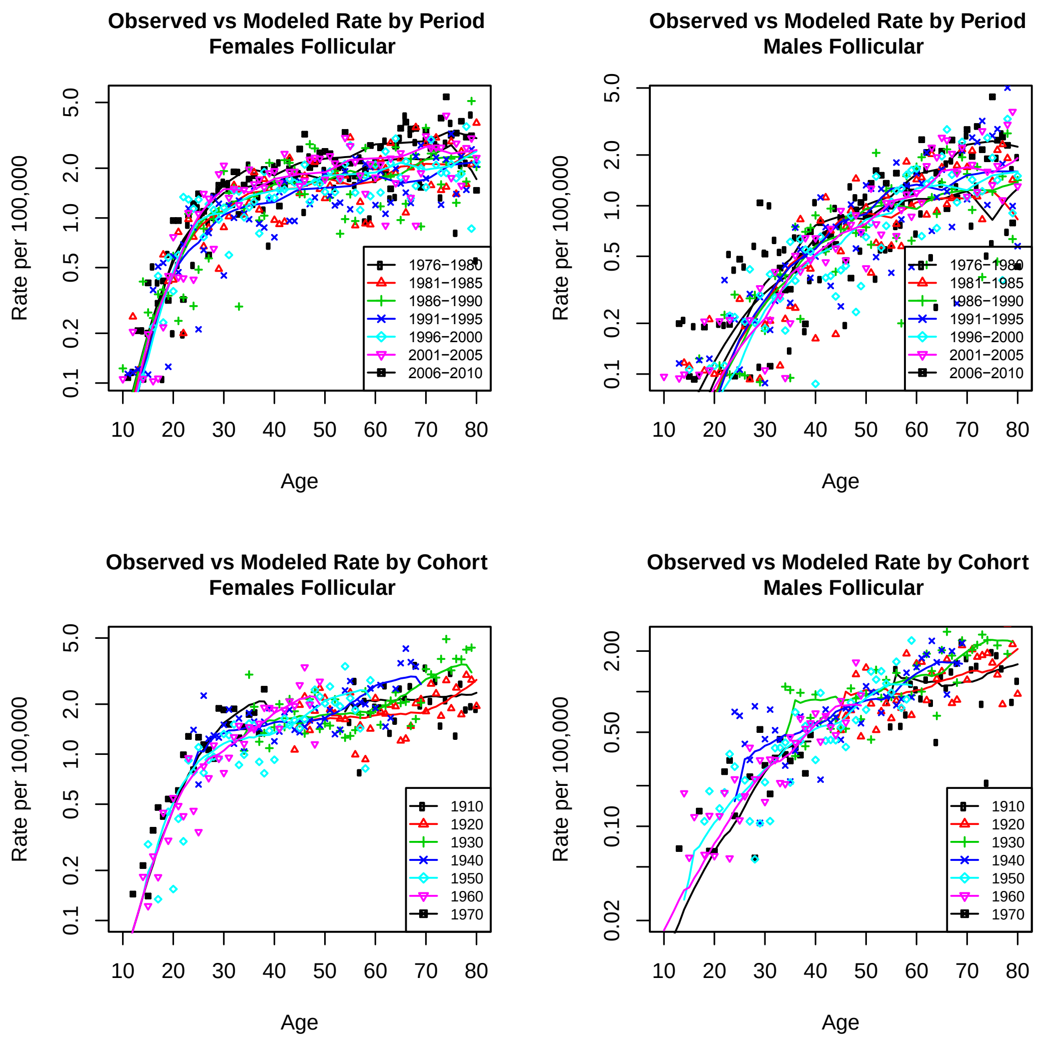

Supplement: Additional file 1: Table S1. — Trends of thyroid cancer incidence by gender, race, histology and stage –Joinpoint analyses 1973-2010. Figure S1. Thyroid cancer incidence period and cohort trends by gender and stage – TSCE-P-C thyroid cancer model. Figure S2. Thyroid cancer age-adjusted incidence rates by gender, race and stage. Table S2. Akaike information criteria (AIC*) values for Age-Period-Cohort models relative to the AC model**. Figure S3. Age effects by race and sex (all cases)- Age-period-cohort models. Figure S4. Age-effects by tumor stage (all cases)- Age-period-cohort models. Figure S5. Thyroid cancer incidence period and cohort trends by gender and race – Age-period-cohort models. Figure S6. Thyroid cancer incidence period and cohort trends by gender and stage – Age-period-cohort models. Figure S7. Observed versus fitted thyroid cancer incidence by gender in Whites. Figure S8. Observed versus fitted thyroid cancer incidence by gender in Blacks. Figure S9.Observed versus fitted thyroid cancer incidence among papillary histology by gender. Figure S10. Observed versus fitted thyroid cancer incidence among follicular histology by gender. (PDF 1164 kb) [file 12889_2015_2108_MOESM1_ESM.pdf]
